# Genomic Evidence of SARS-CoV-2 Reinfection Involving E484K Spike Mutation, Brazil

**DOI:** 10.3201/eid2705.210191

**Published:** 2021-05

**Authors:** Carolina K. V. Nonaka, Marília Miranda Franco, Tiago Gräf, Camila Araújo de Lorenzo Barcia, Renata Naves de Ávila Mendonça, Karoline Almeida Felix de Sousa, Leila M. C. Neiva, Vagner Fosenca, Ana V. A. Mendes, Renato Santana de Aguiar, Marta Giovanetti, Bruno Solano de Freitas Souza

**Affiliations:** D’Or Institute for Research and Education (IDOR), São Rafael Hospital Center for Biotechnology and Cell Therapy, Salvador, Brazil (C.K. Vasques Nonaka, B. Solano de Freitas Souza);; São Rafael Hospital Department of Infectology, Salvador (M.M. Franco, C. Araújo de Lorenzo Barcia, R. Naves de Ávila Mendonça, K. Almeida Felix de Sousa, A. Verena Almeida Mendes);; Fundação Oswaldo Cruz, Gonçalo Moniz Institute, Salvador (T. Gräf);; Coordenação Geral dos Laboratórios de Saúde Pública/Ministério da Saúde, Brasília, Brazil (V. Fosenca);; University of KwaZulu-Natal College of Health Sciences, Durban, South Africa (V. Fosenca);; Universidade Federal de Minas Gerais Instituto de Ciências Biológicas, Belo Horizonte, Brazil (V. Fosenca, R.S. de Aguiar, M. Giovanetti);; Fundação Oswaldo Cruz Instituto Oswaldo Cruz, Rio de Janeiro (M. Giovanetti);; University Campus Bio-Medico of Rome Unit of Medical Statistics and Molecular Epidemiology, Rome, Italy (M. Giovanetti)

**Keywords:** COVID-19, coronavirus disease, SARS-CoV-2, severe acute respiratory syndrome coronavirus 2, viruses, respiratory infections, zoonoses, E484K spike mutation, reinfection, genomic surveillance, Brazil

## Abstract

Uncertainty remains about how long the protective immune responses against severe acute respiratory syndrome coronavirus 2 persists, and suspected reinfection in recovered patients has been reported. We describe a case of reinfection from distinct virus lineages in Brazil harboring the E484K mutation, a variant associated with escape from neutralizing antibodies.

Viral evolution might favor reinfections (*1*), and the recently described spike mutations, particularly in the receptor binding domain in severe acute respiratory syndrome coronavirus 2 (SARS-CoV-2) lineages circulating in the United Kingdom, South Africa, and most recently in Brazil (A. Rambaut et al., unpub. data, https://virological.org/t/preliminary-genomic-characterisation-of-an-emergent-sars-cov-2-lineage-in-the-uk-defined-by-a-novel-set-of-spike-mutations/563; H. Tegally et al., unpub. data, https://doi.org/10.1101/2020.12.21.20248640; C.M. Voloch et al., unpub. data, https://doi.org/10.1101/2020.12.23.20248598), have raised concern on their potential impact in infectivity, immune escape, and reinfection. We report a case of reinfection from distinct SARS-CoV-2 lineages in Brazil harboring the E484K mutation, a variant associated with escape from neutralizing antibodies (*2*; A.J. Greaney, unpub. data, https://doi.org/10.1101/2020.12.31.425021; Z. Liu, unpub. data, https://doi.org/10.1101/2020.11.06.372037). 

A 45-year-old woman residing in Salvador (Bahia State, northeast Brazil) with no underlying conditions had symptoms of viral infection on 2 occasions (May 26, 2020, and October 26, 2020). In the first episode, the patient had diarrhea, myalgia, asthenia, and odynophagia for ≈7 days. She took 40 mg prednisone for 5 days and returned to normal activities 21 days later, after resolution of symptoms without sequelae or complaints. In the second episode, which was symptomatically more severe in terms of intensity and duration, the patient had headache, malaise, diarrhea, cough, and sore throat that evolved to myalgia and ageusia, muscle fatigue, insomnia, mild dyspnea on exertion, and shortness of breath. In both episodes, however, disease was classified as mild, and she was treated at home, not requiring hospitalization. 

The patient was a healthcare executive. Identified workplace exposure included frequent meetings with coronavirus disease (COVID-19) frontline physicians and healthcare teams. Also, before the second episode, she attended a meeting with a group of physicians, one of whom had COVID-19 diagnosed in the days following.

On both occasions, viral RNA was extracted from nasopharyngeal swab specimens and tested for SARS-CoV-2 by multiplex real-time reverse transcription PCR (rRT-PCR) Allplex SARS-CoV-2 assay (Seegene, https://www.seegene.com). Both times, results of rRT-PCR tests targeting 3 genes (N, E, and RdRp) were positive for SARS-CoV-2 (Figure, panel A). Cycle threshold values of N, E, and RdRp targets were 25, 26, and 27 in the first episode and 21, 12, and 17 in the second episode, respectively. In the second episode, the patient had a high viral load (presumed because of low cycle threshold values detected). Four weeks after the patient tested positive by rRT-PCR in the second episode, an IgG test against S1 protein by chemiluminescence (VITROS, Ortho Clinical Diagnostics, https://www.orthoclinicaldiagnostics.com) yielded a positive result. We then sequenced swab specimens by using PGM Ion Torrent (ThermoFisher, https://www.thermofisher.com), according to the manufacturer’s instructions. A total of 1,405,009 mapped reads for sample A (from the first episode) and 2,570,182 reads for sample B (from the second episode) were obtained, resulting in a sequencing mean depth >1,000× for both samples and a coverage of >99%.

**Figure:**
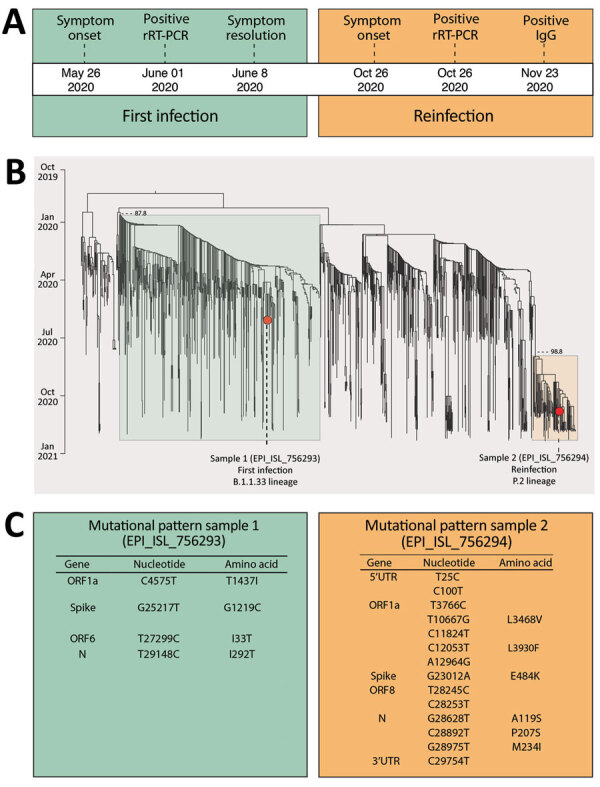
Molecular characterization of a severe acute respiratory syndrome coronavirus 2 reinfection case in Salvador, Bahia State, northeast Brazil. A) Timeline of symptom onset and molecular and serologic diagnosis. B) Time-scaled maximum-likelihood tree, including the new genomes (GISAID accession nos. EPI_ISL_756293 and EPI_ISL_756294; https://www.gisaid.org) recovered from a 45-year-old woman residing in Salvador and full-length viral genomes from Brazil available through GISAID as of January 14, 2021 (Appendix Table, https://wwwnc.cdc.gov/EID/article/27/5/21-0191-App1.xlsx). New genomes are highlighted with red circles. Branch support (SH-aLTR >0.8) is shown at key nodes. C) Mutational pattern of the 2 isolates obtained from the same patient within a 147-day interval. Only unique mutations and lineage defining mutations for B.1.1.33 and P.2 are shown. ORF, open reading frame; rRT-PCR, real-time reverse transcription PCR; UTR, untranslated region.

We further assessed the distinct viral origin of the 2 infections by phylogenetic inference, comparing the 2 new isolates (GISAID accession nos. EPI_ISL_756293 and EPI_ISL_756294; https://www.gisaid.org) with all SARS-CoV-2 genomes from Brazil available through GISAID as of January 14, 2021. Only genomes >29,000 bp and <1% of ambiguities were retrieved (n = 1,164). Sequences were aligned by using MAFFT (*3*) and submitted to IQ-TREE for maximum-likelihood phylogenetic analysis (*4*). We inferred time-scaled trees by using TreeTime (*5*).

Comparison of the phylogenetic profiles of the 2 new sequences with contemporaneous sequences from Brazil (Appendix Table, https://wwwnc.cdc.gov/EID/article/27/5/21-0191-App1.xlsx) clearly demonstrated that the 2 COVID-19 episodes, separated by a 147-day interval, were indeed caused by different SARS-CoV-2 lineages, confirming reinfection (Figure, panel B). In the first episode, the lineage B.1.1.33 was detected, whereas lineage P.2 (an alias for B.1.1.28.2) was detected in the second infection (Figure, panel B), according to the Pangolin lineage classification (https://github.com/hCoV-2019/pangolin [accessed 2021 Jan 11]). Further, we identified several mutations distinguishing the 2 genomes (Figure, panel C), 2 of which were in the SARS-CoV-2 spike glycoprotein. In the first infection, the retrieved genome had the S:G1219C mutation, whereas the mutation S:E484K was observed in the second infection.

This reinfection case aligns with another reinfection recently described in Brazil in which a first infection with the B.1.1.33 lineage was followed by a second one with the P.2 lineage (P.C. Resende et al., unpub. data, https://virological.org/t/spike-e484k-mutation-in-the-first-sars-cov-2-reinfection-case-confirmed-in-brazil-2020/584). The E484K mutation, located in the viral receptor binding domain, has been emerging independently in several SARS-CoV-2 variants, and its monitoring is of pivotal importance in the current stage of the pandemic. At least 3 main lineages harbor E484K: B.1.351, first identified in South Africa and widespread worldwide (H. Tegally et al.); P.1, recently described in Manaus, Brazil, which harbors a constellation of new mutations (including N501Y) (N.R. Faria et al., unpub. data, https://virological.org/t/genomic-characterisation-of-an-emergent-sars-cov-2-lineage-in-manaus-preliminary-findings/586); and P.2, also described in Brazil (C.M. Voloch et al.) and already detected in the United Kingdom, United States, Canada, and Argentina (https://cov-lineages.org/lineages.html). Our report of SARS-CoV-2 reinfection with a E484K variant corroborates in vitro and in silico studies that estimated the potential of lineages carrying this mutation to escape from neutralizing antibodies (*3*; Z. Liu et al.) and highlights the importance of genomic surveillance to detect and monitor the emergence of new viral linages with possible implications for public health policies and immunization strategies.

Appendix Additional information about genomic evidence of SARS-CoV-2 reinfection involving E484K spike mutation, Brazil.
